# *SNCA* overexpression disturbs hippocampal gene expression trajectories in midlife

**DOI:** 10.18632/aging.101691

**Published:** 2018-12-13

**Authors:** Thomas Hentrich, Zinah Wassouf, Olaf Riess, Julia M. Schulze-Hentrich

**Affiliations:** 1 Institute of Medical Genetics and Applied Genomics, University of Tübingen, Tübingen, Germany

**Keywords:** alpha-synuclein, Parkinson’s disease, age, midlife, gene-environment interaction, gene expression analysis, systems biology

## Abstract

Synucleinopathies like Parkinson’s disease and dementia with Lewy bodies originate from a complex and still largely enigmatic interplay of genetic predisposition, age, and environmental factors. While progressively declining motor functions hallmark late-life symptoms, first signs of the disease often surface already decades earlier during midlife. To better understand early disease stages with respect to the genetic, temporal, and environmental dimension, we interrogated hippocampal transcriptome data obtained during midlife for a mouse model overexpressing human *SNCA*, a pivotal gene in synucleinopathies, under different environments. To relate differentially expressed genes to human, we integrated expression signatures for aging and Parkinson’s disease. We identified two distinctive modes of age-dependent disturbances: First, cellular processes seemingly activated too early that reflected advanced stages of age and, second, typical longitudinal adaptations of the system that no longer occurred during midlife. Environmental enrichment prevented both disturbances modes despite persistent *SNCA* overload. Together, our results caution the view that expression changes characterising early stages of *SNCA*-related pathology reflect accelerated aging alone. Instead, we provide evidence that failure to undergo healthy adaptions during midlife represents a second origin of disturbances. This bimodal disturbance principle could inform therapeutic efforts to distinguish between preventive and restorative attempts to target the disease.

## Introduction

The pathology of synucleinopathies such as Parkinson’s disease (PD) and dementia with Lewy bodies (DLB) is characterized by increasing abnormal accumulation and aggregation of alpha-synuclein protein encoded by the *SNCA* locus [1]. Genetic studies further emphasize the role of *SNCA* as point mutations and genomic multiplications are linked to familial forms of PD in a gene dose-dependent manner [2–5]. Genetic *SNCA* defects, however, account for only a small fraction of cases. The majority of synucleinopathies seemingly originates from a complex and still largely enigmatic interplay of genetic predisposition, age, and environmental factors.

While hallmark clinical symptoms of PD and DLB patients are late-life progressive motor impairments, pre-motor phenotypes often surface decades earlier during midlife [6]. Increasing evidence also suggests this time window to be critical for environmental factors to modulate disease unfolding as specifically exercise and physical activity during midlife have been found to lower the risk for PD [7,8] and dementia in elderly [9,10].

Part of early non-motor characteristics of *SNCA*-related pathology are cognitive impairments with respect to memory retrieval and decision making as well as behavioural changes like depression and anxiety [6,11] that are most prominent in synucleopathies with dementia like DLB. As a key brain area for memory formation that is also affected by age-associated memory decline early on [12], the hippocampus is critically linked to these deficits. It also represents a central hub that integrates external cues into the brain sensory circuits [13] and, hence, lends itself to study gene-environment interaction in synucleinopathies. While previous studies have focussed on revealing gene expression changes for advanced stages of age in healthy individuals and Alzheimer’s disease patients [14–16], changes of gene activity in the context of *SNCA*-overexpression—in particular hippocampal changes during midlife—remain to be elucidated. Developing a better understanding as to how genes, age, and environment come together during this time window and influence hippocampal gene activity in context of *SNCA* might, thus, be pivotal to steer much-needed therapeutic opportunities that seek to delay or even prevent further unfolding of pathology in synucleinopathies and improve quality of life for patients.

To this end, we interrogated hippocampal transcriptome data of 6- and 12-month-old wildtype and *SNCA*-overexpressing mice that were housed in different environments. Our analyses identified two distinctive modes of disturbances that were age-dependently induced by *SNCA* overexpression: First, changes in gene activity that seemed to occur too early and reflected advanced age and, second, typical changes that no longer occurred along midlife adaption trajectories of hippocampal gene activity. We further examined these disturbance principles by revisiting our previous finding that environmental enrichment—a housing condition that mimics physical and mental activity for rodent models—largely prevents *SNCA*-induced disturbances [17], and further characterized candidate genes in the age-*SNCA*-dependent interactome that prominently responded to enriched conditions, suggesting a transducer capacity for of beneficial environmental cues onto distinct subsets of disturbed genes.

## RESULTS

### Hippocampal gene expression changes through *SNCA* overexpression occurred age-dependently

**Figure 1:**
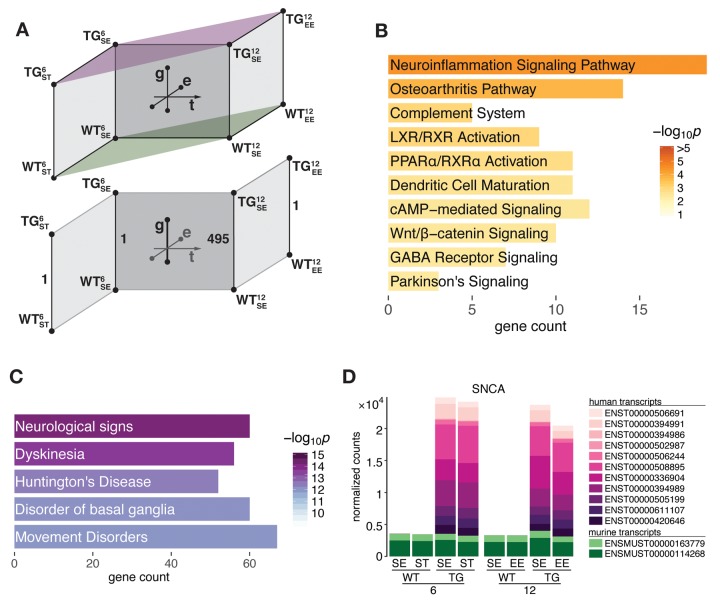
***SNCA* overexpressing mice developed hippocampal transcriptome disturbances in an age-dependent manner between 6 and 12 months of age.** (**A**) Schematic diagram of experimental groups along the genotype (wildtype WT, transgenic TG), age (6 and 12 months), and environment (standard environment SE, enriched environment EE, stress ST) axis that were used to determine differential expression in the hippocampal transcriptome of mice. Lower part highlights age-dependent increase of DEGs in TG animals in SE. (**B**) Overrepresented pathways among 495 DEGs derived for 12-month-old TG mice in SE. Ten most significant terms, their adjusted *p* values, and overlapping DEG count shown. (**C**) Disease aspects and biological functions overrepresented among 495 DEGs. Top five significant terms shown. (**D**) Composition and relative expression levels of murine and human *SNCA* transcript isoforms.

To better understand gene expression changes underlying early stages of *SNCA*-related pathogenesis we interrogated hippocampal transcriptome data of 6- and 12-month-old *SNCA*-overexpressing (TG) and wildtype (WT) mice that were housed in either a standard (SE), enriched (EE), or chronic stress environment (ST) for differentially expressed genes (DEGs) along the genotype (g), time (t), and environment (e) axis (Fig. 1A). With respect to the genotype axis, no DEGs besides *SNCA* were identified in 6-month-old TG mice in both tested environmental conditions (Fig. 1A). In sharp contrast, the number of DEGs for 12-month-old TG mice housed in SE increased to 495, which were overrepresented for several pathways linked to *SNCA* pathology before, most significantly *neuroinflammation signalling* (Fig. 1B, Table S1). These genes were also enriched for disease terms that reflect the pivotal role of *SNCA* in Parkinson’s disease and other synucleinopathies (Fig 1C).

The age-dependent increase of DEGs between 6 and 12 months with respect to genotype cannot be attributed to altered activity of the transgene itself as its (over-) expression level and compositional shares of murine and human transcript isoforms remained virtually identical (Fig 1D). Computational estimates of the underlying cell type composition neither indicated shifts between neuronal and glial cell populations for any of the experimental groups during that time frame (Fig. S1).

Together, these findings suggest an unfolding of events during 6 and 12 months of age that originate from the interference of persistent *SNCA* overexpression and dynamics of the system during midlife that resulted in observed disturbances in TG animals under standard conditions. Intriguingly, these disturbances were not observed when TG animals were housed in enriched conditions (Fig. 1A, lower part), highlighting the beneficial capacity and compensatory effects of the EE in context of *SNCA* overexpression that we have previously described in greater detail [17].

### Midlife changes in hippocampal gene expression occurred largely irrespective of environmental condition

**Figure 2:**
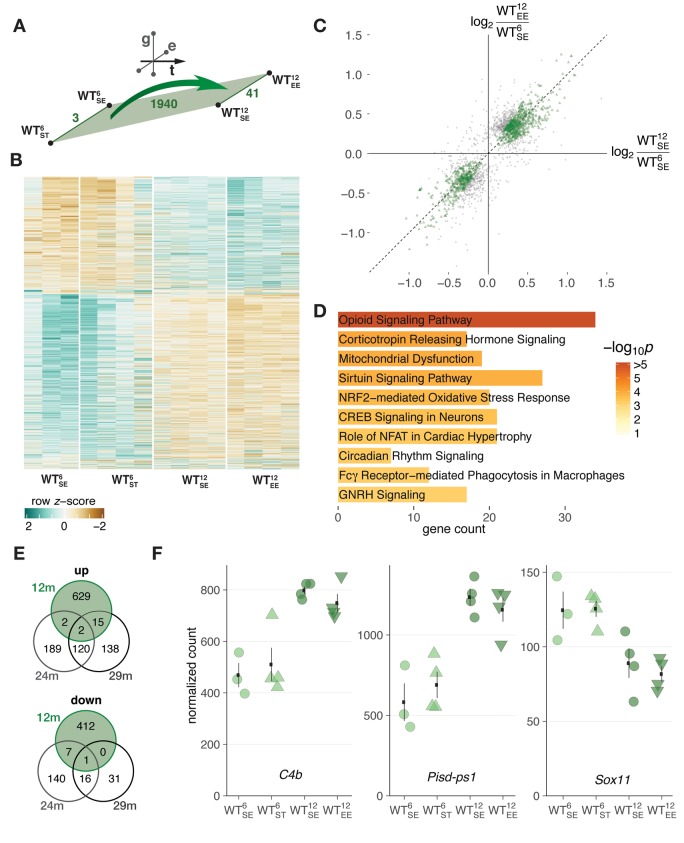
**Midlife aging in WT mice was associated with hippocampal gene expression changes largely irrespective of environmental condition.** (**A**) Schematic diagram showing number of DEGs derived from comparing 6- and 12-month-old WT mice. (**B**) Heatmap of hierarchically clustered *z*-scores for 1940 DEGs derived from comparing 6- and 12-month-old WT mice across environmental conditions. (**C**) Scatter plot of gene expression changes between 6- and 12-month-old WT mice with respect to standard (*x*-axis) and enriched (*y*-axis) environment. 1251 DEGs within the 50% window around the ordinary diagonal coloured in green. (**D**) Enriched pathways for 1251 DEGs from (**C**). Ten most significant terms, their adjusted p values, and overlapping DEG count shown. (**E**) Venn diagrams comparing DEGs identified between 6- and 12-month-old WT mice with known age-regulated genes in the hippocampus of 24- and 29-month-old mice [16]. (**F**) Expression changes of *C4b*, *Pisd-ps1*, and *Sox11* between 6- and 12-month-old WT mice plotted as individual data points with mean ± SEM.

To put dysregulated genes that arose age-dependently in context of *SNCA* overexpression into perspective, we first examined expression changes in WT animals to identify typical changes during midlife. For WT animals, we identified 1940 DEGs that arose between 6 and 12 months of age (Fig. 2A) with consistent and dominant expression changes along the temporal dimension and only modest modulation by environmental condition (Fig. 2B) as reflected by the low count of DEGs (3 and 41 for ST and EE, respectively) along the environment axis (Fig. 2A, Fig. S2).

Genes that changed most consistently along the temporal axis and responded weakest even to the EE were located closest to the standard diagonal when correlating their changes in SE and EE (Fig. 2C, highlighted in green). This subset of genes was enriched for cellular pathways like *opiod signaling*, *sirtuin signaling*, and *CREB signaling in neurons* (Fig. 2D, Table S1). While some of the underlying genes in these pathways have been shown to change expression with age and play a role in age-dependent cellular processes [18–20], collectively these DEGs did not reflect characteristic signatures of aging in the murine hippocampus, indicated by the small overlap with such gene sets (Fig. 2E) [16]. The three DEGs that did overlap were the complement component *C4b*, the non-coding RNA *Pisd-ps1*, and *Sox11* (Fig. 2F), known for its role in development and neurogenesis [19]. Determining the overlap of these DEGs with a second gene set that defines a cross-species age signature based on mouse, rat, and human and is enriched for mainly inflammatory and immune response genes [14], resulted in similarly few commonalities with 5 out of 73 possible candidates.

Collectively, these results suggested that while very first signs of aging began to surface during the investigated time period, the typical spectrum of age-related changes was not yet detectable in 12-months-old WT mice. Instead, expression changes in that period likely reflected normal midlife trajectories and adaptations in gene activity that took place largely irrespective of environmental condition.

### *SNCA* overexpression disturbed midlife gene expression trajectories

**Figure 3:**
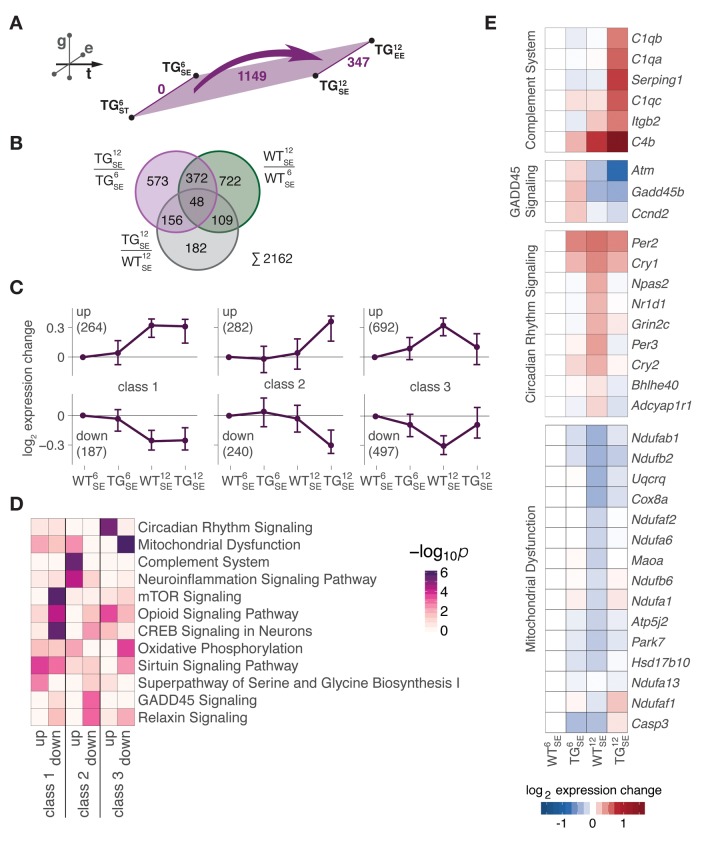
**Midlife gene expression trajectories were disturbed through *SNCA* overexpression in two distinctive modes.** (**A**) Schematic diagram showing number of DEGs derived from comparing 6- and 12-month-old WT (green) and TG (purple) mice*. *(**B**) Venn diagram putting midlife expression changes in TG animals (1149 DEGs in (**A**) in perspective to midlife expression changes in WT animals (1251 DEGs, see Fig. 2C) and *SNCA*-induced disturbances (495 DEGs, see Fig. 1A), totalling to 2162 age-*SNCA*-regulated DEGs. (**C**) Partitioning of 2162 age-*SNCA*-regulated DEGs based on their gene expression pattern in 6- and 12-month-old WT and TG mice in SE (see Fig. S3 for details). Subplots show longitudinal expression medoids and standard deviation of six primary gene clusters grouped into three classes. Number of DEGs per cluster in brackets. (**D**) Canonical pathway analysis for 2162 DEGs according to their cluster/class assignment (see **C**). Two most significant terms per cluster selected, and their significance values across all clusters hierarchically ordered. (**E**) Hierarchically clustered expression changes (relative to WT^6^_SE_) for DEGs attributed to the most significantly overrepresented pathway per cluster in class 2 and 3.

With a better understanding of gene expression changes occurring during midlife in WT animals, we next examined expression changes along the temporal dimension in TG mice. Equivalent to the WT layer, DEGs from all comparisons between 6- and 12-month-old TG animals were combined, totalling to 1149 genes (Fig. 3A). As subsets of these DEGs were also identified along the temporal axis in WT mice and along the genotype axis at 12 months (Fig. 3B), we further explored the indicated interplay between (normal) age-dependent adaptations during midlife and effects of *SNCA* overexpression by partitioning the DEGs into three main classes (Fig. 3C), each comprising two nearly mirror-imaged expression profiles based on WT and TG animals at 6 and 12 months of age (Fig. S3). While class 1 represented genes unaffected by *SNCA* overexpression that had midlife expression trajectories similar to their WT reference, genes in class 2 and 3 clearly showed expression levels for 12-month-old TG mice that seemingly disagreed with the actual age of the animals (Fig. 3C). Specifically, class 3 contained genes that showed adaptations in WT animals but no longer occurred under *SNCA* overexpression. In contrast, class 2 contained genes that had no changes in WT mice during midlife but showed induction/repression under influence of the overexpressed transgene.

When asking for overrepresented cellular processes in these classes, an intriguing pattern emerged (Fig. 3D, Table S1): Changes evoked by the transgene and not typically occurring during midlife (class 2) were highly enriched for pathways like *complement system* and *neuroinflammation signaling* (Fig. 3D), both hallmarks of advanced stages of age [14]. Specifically, all three subunits of the 1q complement component (*C1qa*, *C1qb*, *C1qc*), known to activate the complement system, were prominently upregulated in 12-month-old TG mice (Fig. 3E). *GADD45 signaling*, in contrast, was the most significant pathway among downregulated genes in class 2 like *Atm* and *Gadd45b* that are involved in growth arrest and DNA damage responses (Fig. 3E).

On the other side, class 3 genes, whose trajectories failed to undergo midlife adaptations in context of *SNCA* overexpression, were identified as highly enriched for pathways such as *circadian rhythm signaling* and *mitochondrial dysfunction* (Fig. 3D). Specifically, genes like *Per3*, *Npas2*, and *Cry2* associated in circadian regulation remained below their WT reference levels, while genes attributed to mitochondrial dysfunction such as *Ndufab1*, *Cox8a*, and *Maoa* were higher in TG animals compared to WT mice of the same age (Fig. 3E).

Together, these results suggest a bimodality among age-dependent disturbances in context of *SNCA* overexpression: First, seemingly accelerated cellular processes that are not yet activated in same-aged WT mice (mode I) and, second, disrupted or potentially delayed adaptations of cellular processes that WT animals typically undergo in that phase (mode II).

### Age-*SNCA*-dependent expression changes in mouse agreed with age- and PD-dependent expression signatures in human brain

**Figure 4:**
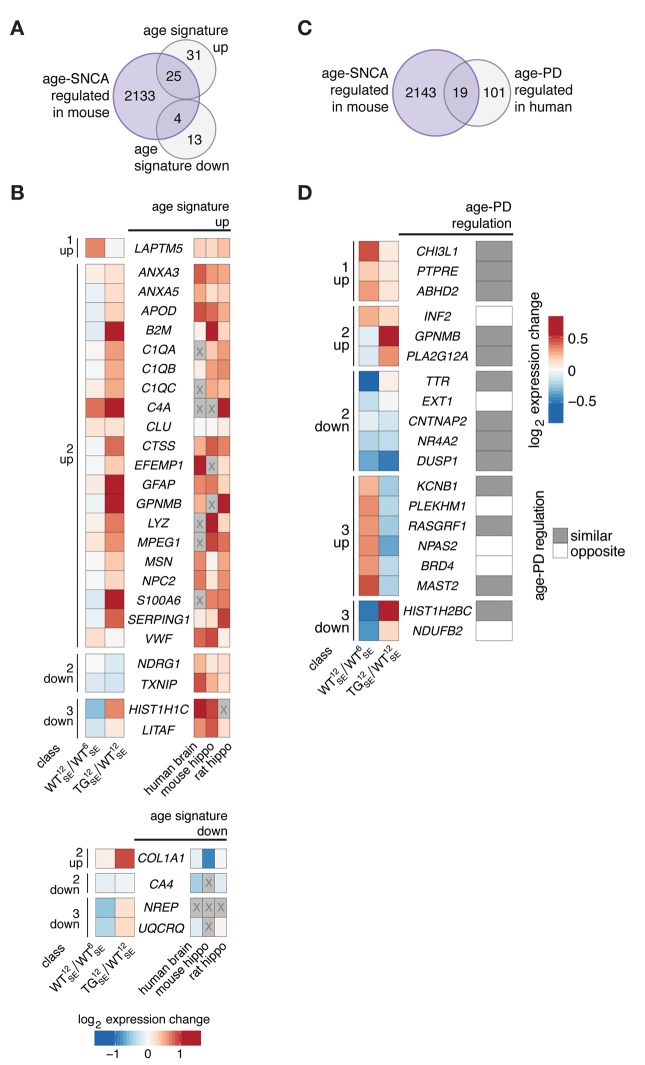
**Age-*SNCA*-dependent expression changes in mouse resembled age- and PD-dependent expression signatures in human.** (**A**) Venn diagram comparing 2162 age-*SNCA*-regulated DEGs (see Fig. 3B) with a cross-species age signature gene set [14]. (**B**) Left panel shows WT^12^_SE_/WT^6^_SE_ and TG^12^_SE_/WT^12^_SE_ expression changes for 29 DEGs grouped by their class assignment that overlap with an age signature gene set (see **A**). Right heatmap reflects age-related expression changes in human brain as well as in mouse and rat hippocampus [14]. Greyed-out cells: no information. (**C**) Venn diagram comparing 2162 age-*SNCA*-regulated DEGs (see Fig. 3B) with genes known to change expression with respect to both age and PD in human [21]. (**D**) Left panel shows WT^12^_SE_/WT^6^_SE_ and TG^12^_SE_/WT^12^_SE_ expression changes for 19 DEGs grouped by their class assignment that overlap with the age-PD signature gene set (see **C**) [21]. Right panel indicates whether age- and PD-dependent expression changes in human are similar or opposite in direction [21].

To put both disturbances modes into perspective, we first used a gene set defining a cross-species age signature of expression changes in mouse, rat, and human brain [14] in order to determine commonalities with age-*SNCA*-dependent DEGs. The overlap between both gene sets, particularly with respect to upregulated reference genes, agreed with the acceleration part of hypothesis that a prominent number of class 2 genes showed expression levels in 12-month-old TG animals that are typically found much later (Fig. 4A, B). Reflected by results on pathway level (Fig. 3D), genes linked to complement system and neuroinflammatory processes such as *C1qa*, *C1qb*, *C1qc*, *C4a*, and *Gfap*, or *S100a6* showed prominent age-*SNCA*-dependent disturbances (Fig. 4B).

In contrast, the overlap of age signature genes with class 3 genes was marginal (Fig. 4B), agreeing with the idea of disrupted and potentially delayed adaptations along midlife trajectories rather than accelerated developments. Evidence for this fundamentally different principle has also been found in human by comparing age- and PD-dependent expression changes [21]. There, too, PD- and age-dependent changes sometimes disagree or even possess opposing directionalities, suggesting that not all PD-dependent expression changes reflect accelerated aging. By comparing age-*SNCA*- with age-PD-dependent DEGs (Fig. 4C), we identified several candidates for which such opposing directionalities between age- and *SNCA*/PD-related changes were consistent across species (Fig. 4D). Among them were *Npas2*, linked to circadian rhythms, and *Ndufb2*, associated with mitochondrial dysfunction (Fig. 4D), both highly enriched pathways in class 3 (Fig. 3C).

These findings indicate that one part of the disturbances through *SNCA* overexpression agree with the shared concept of accelerated aging for some synucleinopathies like DLB and tauopathies, but also derive further evidence for existence of a second disturbance mode in which age- and *SNCA*-related expression trajectories diverge.

### Environmental enrichment largely prevented age-dependent disturbances in the context of *SNCA* overexpression and restored midlife gene expression trajectories.

**Figure 5:**
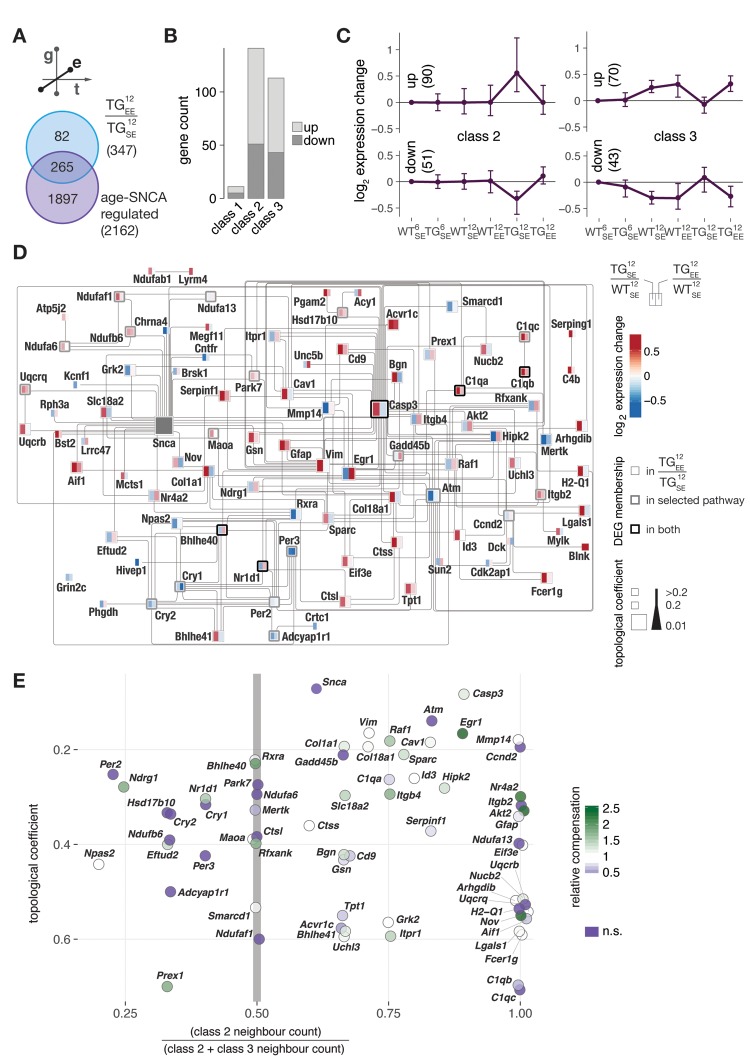
**Environmental enrichment largely prevented age-*SNCA*-dependent disturbances and activated topologically relevant genes in the *SNCA* interactome with respect to both disturbance modes.** (**A**) DEGs identified in response to EE for 12-month-old TG animals compared to 2162 age-*SNCA*-regulated DEGs (see Fig. 3B). (**B**) Distribution of 265 overlapping DEGs (see **A**) with respect to their cluster/class assignments (see Fig 3C). (**C**) Gene expression modulation of 265 DEGs overlapping with class 2 or 3 (254 total) that significantly responded to the EE in TG animals. Expression medoids and standard deviation as well as cluster cardinalities shown. (**D**) Interaction network of DEGs attributed to the most significantly overrepresented pathway per cluster in class 2 and 3 (see Fig. 3E) and their relations to DEGs identified in TG^12^_EE_/TG^12^_SE_. Node colour indicates expression change of a gene in TG^12^_SE_/WT^12^_SE_ and TG^12^_EE_/WT^12^_SE_. Node stroke reflects origin of a DEG, and node size its topological relevance in the network. Edges according to IPA knowledgebase (see Methods for details). (**E**) Characterisations of network genes (see **D**) with respect to their topological relevance and class 2 / 3 neighbourhood ratio. Node colour indicates relative compensation of gene expression change observed in TG animals through provision of EE. DEGs not significantly responding to the EE in TG animals shown in dark purple.

While we have previously shown that environmental enrichment exhibits beneficial impact and is capable of maintaining a near-normal transcriptome state despite persistent *SNCA* overexpression [17], we here revisited these results to put them into temporal perspective. When comparing all 2162 age-*SNCA*-regulated DEGs (see Fig. 3B) with 347 genes responding to the EE in 12-month-old TG mice (Fig. 5A), EE-responsive genes were distributed evenly between class 2 and 3 (Fig. 5B), suggesting the EE to be capable of preventing both modes of disturbances as reflected by the underlying expression profiles (Fig. 5C).

To better understand the EE-response with respect to both disturbance modes, we built an interaction network based on all age-*SNCA*-induced DEGs from the most enriched up- and down-regulated pathways in class 2 and 3 (see Fig. 3E) and their EE modulations (Fig. 5D). Given a gene’s topological relevance in the network, its relative EE response magnitude, and its neighbour count with respect to class 2 and 3, we derived a mapping of network characteristics that allowed identifying topologically relevant and EE-responsive genes (Fig. 5F). Candidates such as *Egr1* and *Nr4a2*/*Nurr1* that have already been suggested in our previous work [17] to play key roles in transducing beneficial environmental cues were found in the top ranks here again. Their location in a class 2-dominated neighbourhood (Fig. 5F) suggested a preferential unfolding of preventive capacity on expression changes that occurred too early in TG animals. On the other hand, candidates such as *Nr1d1* and *Ndrg1* appeared in the class 3-dominated neighbourhood (Fig. 5F), hinting at potential capacities to restore midlife adaptations that failed or were possibly delayed in TG animals. Additionally, candidates like *Bhle40* were connected to equal numbers of class 2 and class 3 DEGs, suggesting them to be capable of transducing beneficial EE effects into both classes.

Taken together, these results point at topologically relevant genes in the age-*SNCA*-dependent interactome that prominently responded to environmental enrichment, suggesting them as pivotal hubs for the unfolding of protection and restoring adaptation trajectories to a near-normal transcriptome state for midlife despite continuous *SNCA* overexpression.

## DISCUSSION

**Figure 6:**
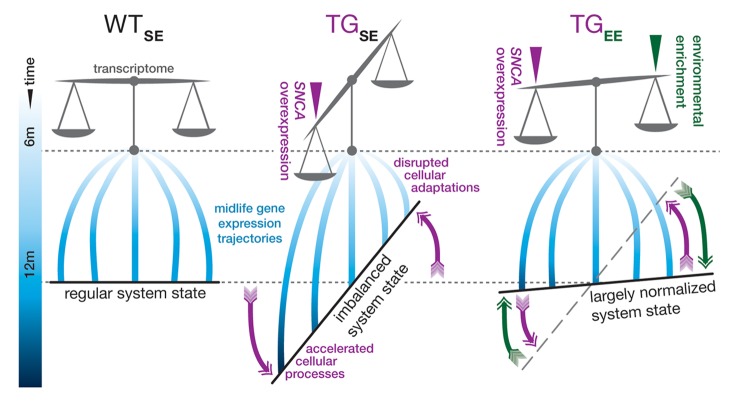
**Graphical summary.** In standard environmental conditions, *SNCA* overexpression in TG mice (TG_SE_) imbalances the hippocampal transcriptome and interferes with gene expression adaptations in midlife so that some cellular processes become accelerated, while others fail to adapt age-adequately in comparison to WT animals (WT_SE_). Provision of environmental enrichment to TG animals (TG_EE_) prevents and counter-balances these disturbances so that a near-normal system state can be maintained despite persistent *SNCA* overexpression.

In this study, we examined expression changes in hippocampal transcriptome data of 6- and 12-month-old wildtype and *SNCA*-overexpressing mice to better understand interactions between the genetic, temporal, and environmental dimension in the unfolding of *SNCA*-related pathology. We identified expression disturbances previously linked to early stages of *SNCA*-related pathogenesis that emerged during midlife and originated from two distinctive interference modes (Fig. 6). Intriguingly, environmental enrichment rendered both modes ineffective, resulting in a near-normal transcriptome state despite persistent *SNCA* overexpression (Fig. 6).

Genes disturbed through the first mode had a prominent neuroinflammatory expression signature, which in healthy individuals and WT mice arises much later in life [14], suggesting *SNCA* overexpression accelerated aging-related processes. This agrees with previous findings that transcriptional disturbances of neurodegenerative diseases resemble aging-associated changes in gene activity [22]. With respect to neuron demise, the PD pathogenesis has been described as a form of accelerated aging [23]. Nevertheless, despite an array of molecular hallmarks shared between aging and PD, including regression of dopamine synthesis [24,25], reduced cerebral density of the type 2 vesicular monoamine transporter (VMAT2) [26], elevated levels of deleted mitochondrial DNA [27], and inflammation [28], it is still of active debate whether PD represents as a form of accelerated aging [28]. While the passage of time is required for PD, aging is not PD [28] as even in elderly of 80+ years, prevalence of the disease is in the 1–2% realm [29].

In contrast, genes suffering from mode II disturbances did no longer show expression changes observed in WT animals during midlife with respect to sirtuin signalling, circadian rhythm, mitochondrial dysfunction, and others. From the perspective of aging as time-dependent progressive decline that renders a living system increasingly vulnerable for diseases and eventually death [30,31], disrupting these trajectories and thereby delaying or halting deteriorating developments might seem advantageous at first glance. Indeed, overexpression of human *SNCA* in *Caenorhabditis elegans* harbours seemingly beneficial capacity as it extends its lifespan [32]. From the perspective of healthy aging, changes mode II genes undergo in WT may not necessarily reflect deviations from a healthy norm but, instead, represent essential systemic adaptions to, for example, bioenergetic or metabolic constraints [33]. In fact, few of these trajectories were influenced by environmental enrichment in WT animals, advocating the idea that associated cellular functions underwent required adaptions of this life span irrespective of environmental condition. From this perspective, disrupting typical midlife adaptation trajectories in the context of *SNCA* overexpression causes failure of the system to adjust adequately.

The circadian rhythm, besides other pathways affected through mode II disturbances, is known to undergo such adaptions also in midlife in human and rodents [34] and is linked to the sleep-wake cycle altered in PD patients [35]. Among clock genes that adapted in WT but failed to do so in TG animals, we found key players like *Per2*, *Per3*, *Cry1*, *Cry2*, and *Npas2*. *Per2* controls *Maoa* that encodes monoamine oxidase and modulates dopamine levels [36]. In agreement, *Maoa* showed mode II disturbances in TG animals, too. Similarly, *Npas2* adaptations were disrupted, in line with altered expression [21] and decreased DNA methylation levels in the *NPAS2* promoter found in PD patients [37]. In fact, hypomethylation of the *NPAS2* promoter has been proposed as an early biomarker for PD as it is also found in peripheral blood [38].

The central role of circadian principles in the cell also extends to mitochondrial biology [39]. In line with mitochondrial dysfunction surfacing early on in idiopathic PD cases [40,41], we observed disruption of several mitochondrial gene trajectories in TG mice. Candidates like *Ndufb2* are reported also for human to have opposite expression directionalities in aging and PD [21], supporting the disruption hypothesis as brain mitochondria normally adapt gene expression and decrease membrane potential as well as electron transfers in complex I and IV with advancing age [42].

Together, both disturbance modes underlying age-*SNCA*-dependent gene expression changes seemingly share the principle of temporal confusions in the unfolding of cellular developments because of *SNCA* overexpression. The resulting disequilibrium of the system, reflected in altered transcriptome state, was largely prevented when *SNCA*-overexpressing mice were housed in enriched conditions. Both aspects allowed further characterisations of potential mediator genes in the EE-induced protection that we have previously put forward [17]. Specifically, we saw evidence for a gradient among potential mediators to preferentially transduce protective environmental cues onto genes from one or the other disturbance mode. Along that gradient, candidates like *Egr1* and *Nr4a2*/*Nurr1* that we have already examined in greater detail [17] were found in an interactome neighbourhood dominated by disturbances through accelerated processes like *complement system* and *neuroinflammation signalling*. In line, *Nr4a2*/*Nurr1* has anti-inflammatory capacity [43], and its elevated expression is known to protect dopaminergic neurons in the midbrain [44]. On the other side of the gradient, in an interactome neighbourhood dominated by midlife adaptation failures, topologically important candidates like *Nr1d1*, also known as *Rev-erb alpha*, could play key roles in preventing *SNCA*-dependent disturbances under EE conditions. Interestingly, its role as a potential therapeutic target has already been suggested before, as improvement of *Nr1d1* expression increases mitochondrial count and content and decreases autophagy flux [45–48]. Near the centre of the hypothesised gradient, genes like *Bhlhe40* were suggested to protectively impact on both disturbances modes, agreeing with its role as an important transcriptional regulator in circadian processes [49] as well as its tight connection to immune cell function [50] and modulating capacities of neuronal excitability and synaptic plasticity in the hippocampus [51].

In summary, the evidence we present for a bimodal partitioning of transcriptomic disturbances that arise in an age-*SNCA*-dependent manner cautions the view that early stages of *SNCA*-related pathology solely reflect accelerated aging. Instead, disruption of healthy adaptation trajectories in midlife seemed to be a second disturbance principle contributing to pathogenic initiations. If further support for this hypothesis can be derived through integrating protein abundancies, physiological parameters, and behavioural phenotypes, candidate genes, their products, or epigenetic makeup could aid in serving as risk biomarkers for PD. In light of inter- and intraneuronal transmission principles that have been proposed as a potential mechanism for pathology spreading induced by overexpressed alpha-synuclein [52], it could be interesting to put our hippocampal observations into perspective with anatomically connected brain regions distant from the site of overexpression. In addition, the validated two-fold disturbance principle could inform efforts towards much-needed therapeutic means for PD to distinguish between accelerated cellular events and disrupted healthy adaptations. Regarding the former, underlying expression changes should probably be approached from a prevention point of view, while the latter might rather be amenable to restorative interventions. For either principle, the plasticity of the midlife transcriptome that we observed in response to environmental enrichment despite stable *SNCA* overload promotes the idea that activable cellular mechanisms have remained at this point and can still counteract initiations of *SNCA*-related pathogenesis.

## METHODS

### Study design and data collection

Hippocampal RNA-seq data sets obtained in 6- and 12-month-old BAC *SNCA* and WT mice that were exposed to either a standard, enriched, or chronic unpredictable mild stress environment were analysed. For details on generation of transgenic mice, environmental enrichment, tissue preparation, and RNA-sequencing please see Wassouf et al. [17]. Raw sequencing data files are available through GEO under accession numbers GSE96961 and GSE116009.

### Quality control, alignment, and expression analysis

Read quality of RNA-seq data in fastq files was assessed using *FastQC* (v0.11.4) [53] to identify sequencing cycles with low average quality, adaptor contamination, or repetitive sequences from PCR amplification. Reads were aligned using *STAR* (v2.5.3a) [54] allowing gapped alignments to account for splicing against a custom-built genome composed of the *Ensembl* *Mus musculus* genome v90 and the human *SNCA* transgene. Alignment quality was analyzed using *samtools* (v1.1) [55]. Normalized read counts for all genes were obtained using *DESeq2* (v1.18.1) [56]. Transcripts covered with less than 50 reads were excluded from subsequent analyses leaving 12,922 genes for determining differential expression in each comparison between experimental groups.

The factorial design of the experiment was captured in a general linearized model defining a gene’s expression (t) as a function of genotype (g), age (a), environment (e), and their interactions. Surrogate variable analysis (*sva*, v3.26.0) was applied to minimize unwanted variation between samples [57]. Given that differences in transcript abundances in brain tissue are often small in magnitude and *in vivo* RNA-seq data are deemed to be more variable [58], we set thresholds of |*log_2_* fold-change | ≥ 0.3 and BH-adjusted *p*-value ≤ 0.1 to determine differential expression.

252 differentially expressed genes (DEGs) showing technical bias (> 4 sd in a sample compared to mean of remaining samples in group) because of sequencing day were excluded. Gene-level abundances were derived from *DESeq2* as normalized read counts and used for calculating *log_2_*-transformed expression changes underlying heatmaps and clusterings that express ratios relative to WT_SE_. Raw counts provided by *DESeq2* also went into calculating nRPKMs (normalized Reads Per Kilobase per Million total reads) as a measure of relative gene expression as motivated before [59]. The *DESeq2* *sizeFactors* served in scaling estimated abundances derived from *Salmon* (v0.8.2) [60] when determining transcript-level compositions of individual genes.

### Gene annotations, pathway and disease associations, and integrations

All Gene ID conversions between mouse and human were done using the *biomaRt* Bioconductor package (v2.34.2) querying v90 of the *Ensembl* database.

Canonical pathways and disease aspects overrepresented among differentially expressed genes as well as their interactions (co-expression, protein-protein interaction, etc.) were derived from *Ingenuity Pathway Analysis* (IPA, v01-12, Qiagen). Further interaction network analyses and visualizations were carried out in Cytoscape (v3.6) [61], ignoring self-loops in topological measures.

Cell type-dependent composition shifts were estimates according to single-cell expression data from the Linnarsson lab [62]. Only relevant cell types in the murine hippocampus were considered.

Analyses regarding directionalities of age-*SNCA*- and age-PD-dependent expression changes were based on (120 mouse orthologues of) a reference gene set obtained in human brain [21].

The age signature reference gene set for human [14] was used to examine signs of accelerated aging among differentially expressed genes in mouse.

## Supplementary Material

Supplementary Figures

Supplementary Table
