# Supplementary material for: SNCA overexpression disturbs hippocampal gene expression trajectories in midlife
Source: Aging (Albany NY). 2018 Dec 13;10(12):4024–41. doi: 10.18632/aging.101691 (PMC6326667; doi:10.18632/aging.101691)
Supplement: Supplementary Figures [file aging-10-101691-s001.pdf]

SUPPLEMENTARY FIGURES

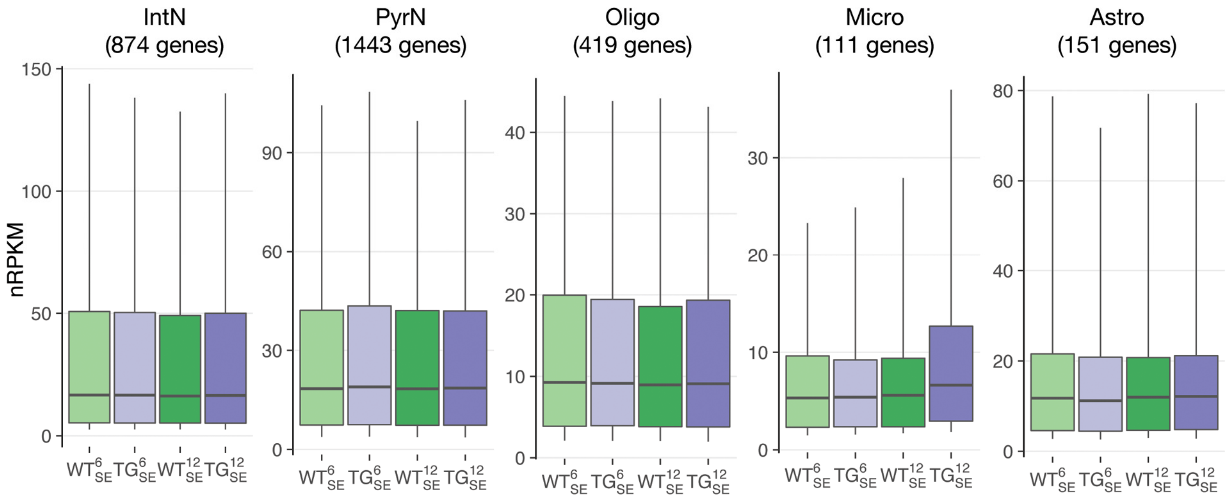

Supplementary Figure 1. Homogeneous cell type composition across genotype and age. Cell type-specific hippocampal gene expression in 6- and 12-month-old WT_{SE} and TG_{SE} animals. Boxplots show geometric mean as well as 10th, 25th, 75th, and 90th quantile of nRPKM values for all genes attributed to distinct cell types based on single-cell data [62]. Number of genes per cell type in brackets: Interneurons (IntN), pyramidal neurons (PyrN), oligodendrocytes (Oligo), microglia (Micro), and astrocytes (Astro). No significant compositional changes were observed (Mann-Whitney U test, two-tailed).

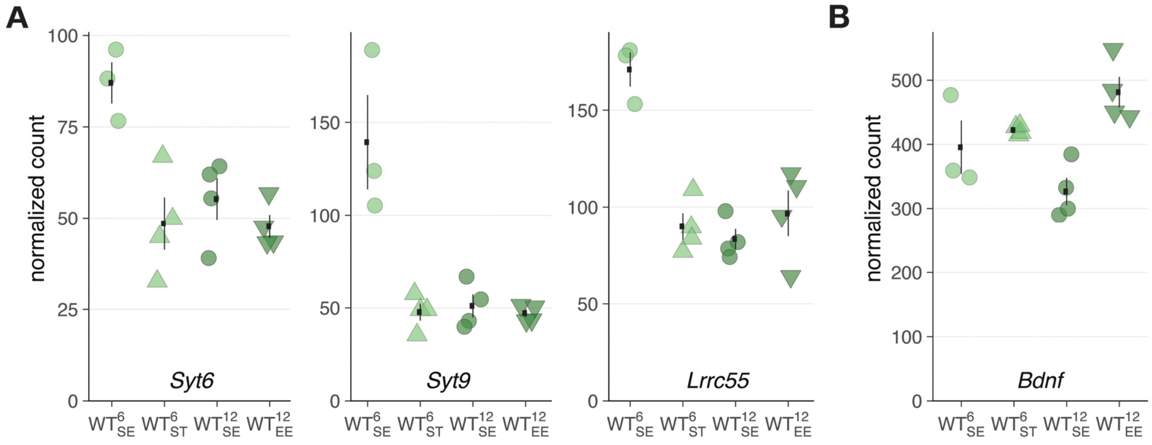

Supplementary Figure 2. Minor effect of chronic unpredictable mild stress on gene expression in wildtype animals. (**A**) Expression levels of three DEGs in WT^{6}_{ST}/WT^{6}_{SE} plotted as individual data points with mean ± SEM. (**B**) Expression level of *Bdnf* plotted as individual data points with mean ± SEM.

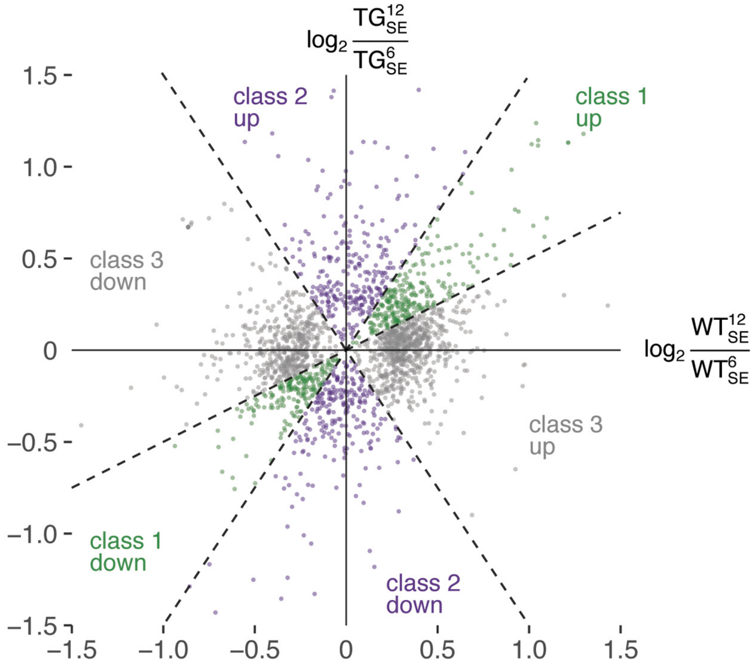

Supplementary Figure 3. Classification of DEGs based on their expression changes between 6- and 12-month-old wildtype and transgenic animals. Scatter plot of gene expression changes between 6- and 12-month-old WT (*x*-axis) and TG (*y*-axis) animals in SE. All 2162 DEGs summarized in Fig 3B are shown. DEGs were partitioned into three main classes such that class 1 represented genes with most similar expression changes for WT and TG animals (a 30º window around the standard diagonal), class 2 with prominent changes in TG animals only (a 30 + 30º window around the *y*-axis), and class 3 with prominent changes in WT animals only (a 30 + 60º window around the *x*-axis).
