# Supplementary material for: SNCA overexpression disturbs hippocampal gene expression trajectories in midlife
Source: Aging (Albany NY). 2018 Dec 13;10(12):4024–41. doi: 10.18632/aging.101691 (PMC6326667; doi:10.18632/aging.101691)
Supplement: Supplementary Table [file aging-10-101691-s002.pdf]

SUPPLEMENTARY TABLE

Supplementary Table 1. Overview of DEGs associated with enriched pathways. Enrichments, significance levels, and associated DEGs for overrepresented pathways shown in Fig 1B, Fig 2D, und Fig 3D.

to Fig 1B

Ingenuity Canonical Pathways	-log(p-value)	Genes
Neuroinflammation Signaling Pathway	4,46	B2M, CASP3, BDNF, TNFRSF1A, ACVR1, MFGE8, MAPK11, FGFR3, TGFBR2, SLC6A11, GABRG2, SLC6A1, PTGS2, GABRD, SNCA, ACVR1C, ACVR2A, IRAK2, PLA2G12A
Osteoarthritis Pathway	3,78	EPAS1, FRZB, CASP3, TNFRSF1A, ANXA2, FGFR3, TGFBR2, GLIS2, SMO, SOX9, PTGS2, IL1RAPL1, FZD2, RBP4
Complement System	3,00	C4A/C4B, SERPING1, C1QA, C1QC, C1QB
LXR/RXR Activation	2,99	C4A/C4B, LYZ, TNFRSF1A, SERPINF1, IL1RAPL1, PTGS2, RXRA, RBP4, APOD
PPARα/RXRα Activation	2,82	TGFBR2, PLCD1, CHD5, PDIA3, ADCY1, ACVR1, SLC27A1, IL1RAPL1, RXRA, ACVR1C, ACVR2A
Dendritic Cell Maturation	2,58	B2M, COL1A2, PLCD1, FGFR3, COL1A1, PDIA3, TNFRSF1A, FCER1G, STAT2, COL18A1, MAPK11
cAMP-mediated signaling	2,50	SMPDL3A, GPR17, RGS2, VIPR1, PDE7B, GRK2, ADCY1, RGS4, RAPGEF3, PKIA, RGS12, CRHR1
Wnt/β-catenin Signaling	2,45	TGFBR2, FRZB, APPL2, ACVR1, SMO, SOX9, SOX8, FZD2, ACVR1C, ACVR2A
GABA Receptor Signaling	2,41	CACNA1I, SLC6A11, GABRG2, ADCY1, SLC6A1, CACNG7, GABRD
Parkinson's Signaling	2,38	CASP3, MAPK11, SNCA

to Fig 2D

Ingenuity Canonical Pathways	-log(p-value)	Genes
Opioid Signaling Pathway	9,06	RAP1B, NOS1, RPS6KA3, RGS12, GNG7, GRINA, RGS17, AP1G2, GNG11, AKT1, ARRB1, CAMK2A, GRIN2C, ADCY5, RPS6KB2, ATF4, CACNG7, RYR1, RPS6KA2, PPP3CA, MAP2K7, CACNB1, GNAS, PRKCQ, GRK3, EGR4, CACNB4, RGS4, RAP1A, PRKCG, ARRB2, PRKAR2B, CLTA, RGS11
Corticotropin Releasing Hormone Sig.	4,07	RAP1B, NOS1, CACNB1, PRKCQ, GNAS, CACNB4, ARPC5, MAPK11, RAP1A, PRKCG, JUN, PRKAR2B, ADCY5, CACNG7, ATF4, JUND, NPR2
Mitochondrial Dysfunction	3,91	HSD17B10, MT-ND6, UCP2, COX8A, TRAK1, MT-ND4L, NDUFA1, NDUFB3, NDUFA13, GSR, MT-ND5, PARK7, UQCR10, ATP5J2, NDUFB6, NDUFAB1, NDUFB2, UQCRQ, MAOA
Sirtuin Signaling Pathway	3,88	POLR2F, TIMM13, GADD45B, GADD45G, PAM16, NDUFA1, SIRT4, NDUFB3, SOD3, AKT1, JUN, CRTC2, SIRT6, NDUFB6, NDUFAB1, TIMM8A, MT-ND6, EPAS1, UCP2, SLC2A1, GABPB2, DOT1L, TIMM8B, MT-ND4L, NDUFA13, MT-ND5, NDUFB2
NRF2-mediated Oxidative Stress Resp.	3,67	MAP2K7, PRKCQ, GSTM5, DNAJC9, JUNB, DNAJC15, DNAJB9, DNAJA1, SOD3, PRKCG, GSR, FGFR3, JUN, AKT1, ABCC1, ATF4, JUND, AOX1, TXN, UBE2E3
CREB Signaling in Neurons	3,55	CACNB1, POLR2F, GNAS, PRKCQ, PDIA3, GRID2, CACNB4, GNG13, GNG7, PRKCG, FGFR3, GRIK5, AKT1, POLR2A, PRKAR2B, GNG11, CAMK2A, GRIN2C, ADCY5, ATF4, CACNG7
Role of NFAT in Cardiac Hypertrophy	3,36	AKAP5, CACNB1, MAP2K7, GNAS, PRKCQ, PDIA3, CACNB4, GNG13, MAPK11, GNG7, PRKCG, CABIN1, FGFR3, AKT1, PRKAR2B, GNG11, CAMK2A, ADCY5, HDAC7, CACNG7, PPP3CA
Circadian Rhythm Signaling	3,32	PER3, NR1D1, GRIN2C, CRY2, ATF4, PER2, GRINA
Fcg Receptor-mediated Phagocytosis	3,25	PLA2G6, PXN, TLN2, PRKCQ, AKT1, ARPC1B, ARPC5L, VAV3, ARPC5, RPS6KB2, INPP5D, PRKCG
GNRH Signaling	3,18	CACNB1, MAP2K7, PXN, GNAS, PRKCQ, PAK6, CACNB4, MAPK11, GNG7, PRKCG, JUN, PRKAR2B, GNG11, CAMK2A, ADCY5, CACNG7, ATF4

to Fig 3D

**Class**	**Ingenuity Canonical Pathways**	**-log(p-value)**	**Genes**
class 1 up	Sirtuin Signaling Pathway	3,63	SCNN1A, MT-ND5, MT-ND6, UCP2, MAPK4, MT-CYB, GTF3C2, MT-ND4, MT-ND4L, SOD3, SIRT4
	Serine/Glycine Biosynthesis I	2,67	DUSP26, SHMT2
class 1 down	mTOR Signaling	5,7	RPS19, AKT2, PRKCD, RPS18, RPS6KA3, FGFR2, RPS21, PRKD3, AKT1S1, RPS14
	CREB Signaling in Neurons	4,48	POLR2F, AKT2, PRKAR2B, PRKCD, GRID2, CACNG5, CACNB4, FGFR2, ATF4, PRKD3
class 2 up	Complement System	5,48	C4A/C4B, ITGB2, SERPING1, C1QC, C1QA, C1QB
	Neuroinflammation Signaling Pathway	4,25	TGFBR2, B2M, HMOX1, GABRG2, TNFRSF1A, TREM2, TYK2, GABRB1, ACVR1, ACVR1C, SNCA, GABRA2, PLA2G12A
class 2 down	GADD45 Signaling	3,11	CCND2, GADD45B, ATM
	Relaxin Signaling	3,04	GNG4, FOS, PDE7B, RXFP1, ADCY1, GNB5, ATM
class 3 up	Circadian Rhythm Signaling	5,3	ADCYAP1R1, BHLHE40, CRY1, CRY2, GRIN2C, NR1D1, PER2, PER3
	Opioid Signaling Pathway	3,5	ADCY5, AKT1, ARRB1, ARRB2, CACNB1, CACNG7, CAMK2A, GNG7, GRIN2C, GRK2, GRK3, MAP2K7, NOS1, PRKCG, RGS12, RPS6KA2, RPS6KB2, RYR1
class 3 down	Mitochondrial Dysfunction	5,32	HSD17B10, NDUFAF1, CASP3, COX8A, NDUFAF2, NDUFA1, PARK7, NDUFA6, ATP5MF, NDUFB6, NDUFAB1, UQCRQ, NDUFB2, MAOA
	Oxidative Phosphorylation	2,97	NDUFA6, COX8A, ATP5MF, NDUFB6, NDUFA1, NDUFAB1, UQCRQ, NDUFB2
